# A Complete Treatise on Hypochondria

**Published:** 1845-10

**Authors:** 


### ART. VII.

1. *Traité Complet de l'Hypocondrie.* PAR J. L. BRACHET, Professeur de Pathologie générale, President de la Société de Médecine de Lyon, &c. —*Paris et Lyon,* 1844.

*A Complete Treatise on Hypochondria.* Par J. L. BRACHET, Professor of General Pathology, &c.—*Paris and Lyons,* 1844. 8vo, pp. 740.

2. *Traité Pratique, Dogmatique, et Critique de l'Hypocondrie.* Par C. F. MICHÉA, Docteur en Médecine de la Faculté de Paris.—*Paris,* 1845.

*A Practical, Dogmatic, and Critical Treatise on Hypochondria.* By C. F. MICHÉA, M.D. of the Faculty of Paris.—*Paris,* 1845. 8vo, pp. 486.

HAD these works reached us at the same time we should have classed them together in one general notice. This not having been the case, we must now give a distinct account of each.

I. M. Brachet's work, as the title-page informs us, was crowned by the Royal Academy of Medicine of Paris. We may, therefore, *a priori,* assume that it has some merit; not forgetting, however, that the critical verdicts, *pro* and *con,* of academies and royal institutions, have sometimes widely differed from those of the public and of posterity. The author's treatise, being elaborate and carefully composed, affords us a suitable occasion for bringing before our readers the subject to which it is devoted. Hypochondria must be allowed to occupy a tolerably prominent place among the vexing and vexed questions of medical science; and may be said to derive a new interest from the circumstance, that in the nature and phenomena of hypochondria, as well as of hysteria, may be found a solution, in some degree at least, of the perplexing facts of mesmerism.

The disease is described, though without a name, by Hippocrates (and with tolerable accuracy) in the second book of his Treatise de Morbis. Galen also gives a sketch of it, in the form of an extract from the works of Diocles of Carystia. But both Hippocrates and Diocles omit some striking peculiarities of the disease as it manifests itself now-a-days, and more especially in this country: and neither attempt to fix, with any precision, the seat or seats of the affection. Galen attributes it to a viscid condition of the blood and to black bile, and notices the gastric derangements, as well as those "*circa* ventriculum;" and his notions have influenced, till a very recent period, his successors in the art. For centuries past, disorders of the liver and spleen, one or both, have been regarded as the peccant causes of the malady.

Aetius appears to have been the first who announced the doctrine which now prevails, that the disease originates in the brain. Holding, as he did, with force, the humoral pathology, he supposed that while the blood in general was *deteriorated,* that of the brain was absolutely *altered* in hypochondria. But he seems also to have believed (and the opinion is plausible) that the disease might have a stomachic origin, since he speaks of a humoral air, rising from the stomach and affecting the brain by the medium or channel of the pneumogastric nerves. Avicenna and, later, Sennert, who both treat of the disease, viewed the stomach as the site of it; and Vieussens, in his 'History of Internal Diseases,' holds doctrines little different from those of Sennert.

In 1667-70, Willis and Highmore had a controversy on the subject of the pathology of hypochondria, but we shall spare the reader the somewhat idle details of it, nor shall we notice the opinions (in great part mere repetitions of the preceding ones) of Marcucius, Lange, Chatelain, Rondelet, &c. Stahl, in regard to hypochondria, seems to have leant to the humoral pathology, which he repudiated in general; agreeing with the punning enunciation of the ancients, that the "vena *portæ,*" was the "*porta* malorum hypochondriacorum." Boerhaave's opinions are fanciful, nor merit the time or space of a lengthened description. Sydenham confounded hypochondria with hysteria. Willis, among the moderns, first announced the doctrine that hypochondria is a nervous disease; but in this, as we have already remarked, he was anticipated by Aetius, from whom also he seems to have borrowed the notion that some morbid change of the blood in the spleen induced the peculiar affection in the brain and nerves, constituting hypochondriasis. Passing over the theories of intermediate and less important authors, we come to those of Lonyer-Villermay, who, while recognizing the affection of the cerebral organ and of the intellectual and moral faculties, conceived the origin and seat of hypochondria to be in the medulla spinalis and the organic nerves. Georget, first, and subsequently Falret and Dubois, endeavoured to show that the disease had its "local habitation" *in the brain,* though the received nosological nomenclature suggested a different one. Under the name of "*cérébro-pathie*" Dubois affirmed hypochondriasis to be "an essential and idiopathic affection of the brain;"—and this is the last definition of the malady which we intend to notice.

We imagine that much of the doubt and dispute connected with the pathogeny and pathology of hypochondria would be put an end to, by a few simple observations by which the subject might be disentangled from some difficulties and obscurities, in which it has been hitherto unnecessarily involved. Two very important points, for example, in regard to which we must endeavour to have clear ideas, are, first, what exactly is the condition, or rather the congeries of phenomena, which constitute hypochondria; secondly, what is meant by the allegation (founded on what appears to be the right pathology of the disease) that hypochondria is invariably a cerebral affection. On these two points we shall make a few remarks.

Hypochondria is not, in any case, a simple or single affection. It is, on the contrary, emphatically a complex one. Three principal orders of phenomena are easily recognizable: first, lesions of the intelligence or the imagination, namely the greatly exaggerated, or (if it be not a solecism to say so) the fanciful feelings and sensations of which the patient supposes himself to be the subject. The second order are those of morbid sensation, and are distinguished from those of the first order, in being apparently *really* felt, though from causes and in a degree different from what would be the case in almost any disease but the one under consideration. The third order of phenomena consists in derangements, not of the sensory, but organic nerves, by which are produced disorders, chiefly functional, in the corresponding organs. Now, without the presence of *some,* at least, of each of these three classes of symptoms, we cannot have hypochondria. For it is observable, that each of the three classes of symptoms may manifest itself separately. Thus, morbid sensations of the most remarkable kind may be present, but without the slightest mental affection of that peculiar sort known as the hypochondriacal. Again, it is notorious, that the greatest functional disorders of the abdominal or thoracic organs may exist, without any abnormal affection either of the physical or of the moral senses, or of the intellectual powers. Lastly, a monomaniacal aberration in regard to the health or the state of the body may have place, yet without any corresponding error of the nerves of sense, or any functional derangement either of the organic nerves or the organs supplied by them. Thus a man imagines himself to have a limb of glass, but, in all other points, eats, feels, and conducts himself like other men. Hence it appears, that, so to speak, the component parts of hypochondria may and often do exist separately; but it is the *union,* the *simultaneous existence* and conjunction of these parts which constitutes hypochondria.

We shall now instance, very briefly, one or two of the most striking symptoms of the three different orders of phenomena. Of the first order, or those which consist in lesion of the imagination or the intellectual faculties, may be instanced the disproportion of the patient's anxiety about himself, to the frequently unimportant nature of his ailments; the tenacity with which his attention is fixed on his own case; his morbid aptitude to fancy himself the victim of every new disease or symptom which he either reads of, or from which he hears that others are suffering; his propensity to dwell on his complaints; to speak of them to every one; and, in describing them, to employ the most vivid and strong language; and lastly, the *melancholia*, unsociality, and numerous and indescribable whims of manner and idea, which characterize the hypochondriac patient.

The second order of symptoms, or those which consist in lesions of the sensible nerves, are pains of various kinds, and in all parts of the body; feelings of heat and cold; sensations giving rise to a suspicion in the patient's mind, that there are live animals in his stomach, in his chest or abdomen, or under his skin, or in his head, buzzings or explosions in the ear, &c., occasionally great sensibility of the skin.

The third order of symptoms, those namely of an organic site, though generally only functional, consists of pulsations in various parts of the body, which are not to be ranked among *imaginary* sensations, being at least often appreciable by the ear or hand of the practitioner; the occasional slowness, or extreme depression of the heart's action; the vitiation not unfrequently of secretions, as of the mucus or bile; the unusual viscidity of the former; the presence of the same quality along with the almost melanotic appearance in the latter; derangements in the condition of the alvine and urinary discharges, the former of which are often either almost suppressed, or, though liquid, scanty; the latter often presenting the character of hysterical urine.

It thus appears that there is, in hypochondria, present, at the same time, first, moral and intellectual disturbance; secondly, an affection of the sensible nerves, which seems to consist in a morbidly increased sensibility. Of the morbid sensations, thence resulting, the patient is evidently conscious; they become a distinct subject of his intellectual perception; since he comments on them, describes them, often very graphically; although we have every reason to believe, that, whether owing to the peculiar state of the patient's imagination, which prompts him to exaggerate his feelings, or to the exalted sensibility of the nerves of sensation, the causes producing the sufferings of which he complains, would, in persons, *not hypochondriac,* attract incomparably less attention. Lastly, there is disturbance of the function of the organic nerves, since, without organic change and without inflammatory symptoms, there is often long-continued, though it may be, not very marked or serious disorder of the stomach and bowels, the heart, the bladder, &c. In regard to the cerebral phenomena, M. Brachet observes:

"Nothing, doubtless, merits greater attention, than the study of these phenomena. They are indispensable to constitute hypochondria. Without them, all pains the most acute, all sensations the most extraordinary, would not be sufficient to constitute it. That aberration of the morbid imagination must be present, which not only receives painful sensations, but which interprets and draws inferences from them through its own peculiar pathological prism, and which, in its ill-founded fears, gives them the dark hue which itself has, and makes out of them as many grave maladies as the mind can entertain."* (p. 477.)

The second point, which we proposed to consider, is, in what sense and to what extent it is true that hypochondria is always a *cerebral* affection. The affirmation, that hypochondria is always a cerebral affection, and the accuracy of which, we conceive, is indisputable, is yet widely different from the allegation, that the *train* of symptoms out of which, in their progress and complications, hypochondria ultimately results, of neces-

* We translate literally, but are not accountable for the incorrect expression of the author.

sity *begins* in the brain. As an *universal* rule, this doctrine is as inaccurate as the former is the contrary. A few observations will, we apprehend, place this in a clear light.

Cases such as the following frequently present themselves. A person has long laboured under stomachic, intestinal, or urinary disease, or suffered from some irregularity in the action of the heart, as for example, intermitting or slow pulse, or from anormal cardiac or arterial pulsations. The patient, during a length of time, exhibits no morbid solicitude about himself or his ailment; but possibly he puts himself, or is put, on a system of physic or diet, and induced to make changes in his habits of exercise, &c., by which as well, it may be, as by his disorder, his general health begins to suffer. Now, at length, hypochondriacal symptoms, indicating that the brain, in common possibly with other organs, has at last been involved, begin to manifest themselves. In this case, though the cerebral affection was obviously necessary to constitute hypochondriasis, till which occurrence the affection, however various, could not possibly be so designated, yet it is plain that the cerebral affection was secondary.

But M. Brachet goes a step further than we have now done, and maintains that even in cases when the first morbid movements in the train of those which issue in hypochondriasis, take place *through* the brain, this organ itself is yet, after all, not primarily but only secondarily affected. We shall allow him to explain himself in his own words:

"We meet with," he observes, "in Dr. Falret (on Hypochondria and Suicide) with the same 'ideas' "—the author has just been quoting from Georget—" the same reasonings; of course the same objections are to be made. We still ask; are these causes moral. . . . . . . . It is evident that, in these observations, it is necessary to torture facts in order to prove these causes to be exclusively in the brain. Mental occupation in the 'study' (travaux de cabinet), a sedentary life, excesses at table, may doubtless predispose to and even cause hypochondria; but most frequently it is not by acting directly on the brain, at the outset. We go further: we say that in three fourths or more of the cases, in which chagrin, ungratified passions, have exerted on the brain an action the most powerful and the most direct, it is not by the encephalon, that is to say, by the derangement of the intellectual faculties or the imagination, that the malady commences. Always or almost always, it does not revert to the brain, save by a consecutive reaction.* Let M. Falret, if he pleases, follow the thread of phenomena, and he will perceive that things occur differently from what they would do, were the brain the organ primarily diseased. Yes! such is the fact! the cause of the malady has acted on the brain, but it has not occasioned hypochondria; chagrin, for example, has diminished the activity of the circulation, has caused a sanguineous concentration on the central organs of circulation and respiration, and has given rise to painful congestions which render sobbing necessary in order momentarily to give impulse to the respiration and circulation. It has further acted on the apparatus of digestion and principally on the stomach. It has lessened the activity of that organ; the appetite is lost, digestion is bad, and stomach-aches or a gastralgia with all its consequences are the result. It is from this assemblage of phenomena or rather from the lesions of which they are the effects (? actes) that result the reactions which influence the brain anew, causing it to experience sufferings, and changing its manner of receiving impressions and of reasoning on its sensations. Further, as we have already said, to say that a disease is necessarily cerebral, because the cause has acted on the

* We translate the author exactly.

brain, would be equivalent to saying that a pneumonia was a disease of the skin, because the cold air which caused it acted primarily on that tegument." (pp. 248-9.)

We, for our parts, cannot see any objection to the view here taken by M. Brachet. We every day meet with cases of dyspepsia, with functional affections of the heart, such as irregular pulse, palpitation, &c., which are clearly traceable to mental excitement or chagrin, and which consequently *must* have been called into existence *through* the brain: yet that organ, in the first instance or perhaps throughout, shall manifest no symptom, either physical or intellectual, of local derangement, or suffering. In such circumstances, a man will tell you that he has had great anxiety about such and such a matter, and that in consequence his stomach and not his brain, is very much out of order. If you question him, he will explicitly tell you, he has pain or weight at his epigastrium, and if you examine his tongue, stools, urine, &c., you will probably find additional evidence of the accuracy of his statement. He will make no complaint whatever of his *head,* and if so, what right have you to presume derangement or suffering to be there?

In stating the above view, we must not be at all understood to maintain, that hypochondria always, or even frequently, originates in the stomach, or in any other organ besides the cerebrum. On the contrary, while we strictly hold that the affection of the latter organ is *indispensable* to constitute hypochondria, we likewise incline to the belief that more frequently than otherwise the disease also *commences* there.

Having discussed the foregoing topics, which, except in so far as they may facilitate the diagnosis, may be viewed as of little practical importance, we now proceed before passing into the therapeutics of the disease, to consider the author's account of the causes, symptoms, and progress of hypochondriasis.

*Causes.* These are either original or acquired. A highly nervous temperament, whether hereditary or induced, conjoined to a natural or accidental *ataxy* of the biliary apparatus, seems to be the one most particularly disposed to hypochondria. In producing the state of the systems, nervous and biliary, now described, (if not constitutional,) all causes calculated at once to stimulate and exhaust the nervous power, will concur; these causes may be moral or physical, or both; and consist of violent passions, or long continued harassment or chagrin; violent or prolonged mental labour; diseases of various organs, as of the stomach, heart, and as we have said, of the liver; long-lasting pain. Sexual excesses and spermatorrhœa are frequent causes.

In forming the diagnosis, so far as regards the *causes,* between hypochondria and other somewhat-resembling complaints, it may be useful here to notice that melancholia presents, in general, less evident *physical* causes than the disease in question; that hysteria (for hypochondriasis occurring in the female, though much less frequently than in the other sex, may be confounded with the affection just named) is generally connected with uterine irregularity; and that gastralgia is very often caused by obvious abuses of food and drink, and generally curable by a strict reformation of these.

*Symptoms.* We have already referred, generally, to some of the most remarkable of these, and shall not recapitulate them. We omitted to notice the frequency of borborymi and flatulent distension of the bowels. In forming one diagnosis from symptoms, we must keep in mind, that the sufferer from melancholia is distinguished by much greater reserve as to his complaints than the hypochondriac: that the complaints of the former rather regard others than himself; that the former has a much stronger disposition to suicide than the latter: and that while the former shuns, the latter courts the aid of the physician.

The pure gastralgic or dyspeptic patient, besides presenting, in general, obvious signs of gastric disorder, such as epigastric pain, eructations, a foul tongue, want of appetite, &c., exhibits no mental dispondency nor any anxiety about the gravity or issue of his disease, beyond what is quite corresponding with the inconveniences he may suffer from it.

The progress of hypochondria is usually gradual. Recovery where the causes are constitutional is rare. The issue is seldom fatal, and when death ensues, it is often difficult or impossible to trace it to hypochondria, or to be able to show that the chances of life were materially affected by that disease. Nay, on the contrary, it is almost proverbial that hypochondriac patients are long-livers. If the remark has any foundation in truth, it must be accounted for from the unusual care which such patients take of themselves, and the readiness with which they adopt the medical measures suggested to them. Sometimes, however, from the exclusiveness and intentness of their attention to their own case, *dementia* is induced, or else some organic affection of the brain or heart, leading to a fatal result.

*Seat of hypochondria.* This is in the nervous system and in the three several parts of that system: namely, in the intellectual and moral organs; in those portions of the brain which minister to sensation; and lastly in that part of the nervous system which regulates organic life. To a great extent, if not wholly, the intellectual and moral portions of the brain are the seat of melancholia, the nerves of sensation and of organic life not being implicated; and in idiopathic gastralgia or dyspepsia, the stomach is of course the exclusive seat of lesion, the affections of remoter organs being in the first instance merely sympathetic, though, of course, they may ultimately become independent and organic. In this way, indeed, as we have already noticed, hypochondria may be grafted on dyspepsia and on other diseases.

The *prognosis* may be founded on the account given of the progress of hypochondria. It is favorable in so far as a *fatal* result is seldom to be apprehended; but unfavorable as far as regards a radical cure.

The *pathology* of hypochondria is, so far as morbid anatomy is concerned, extremely unsatisfactory. Indeed it may be said that, up to the present time, there is not a single structural change in a tissue or organ, which has been clearly identified as peculiar to hypochondriasis, whether as a cause or an effect; for the occasional morbid changes in the heart, the stomach, liver, spleen, brain, with which the disease is sometimes complicated are so various and so inconstant,—so different in different cases, and so often absent in others,—as to show that, between them and the disease, there is no necessary but only an incidental connexion. Georget and Falret indeed, who, both physicians in hospitals for the insane, biassed probably by the predominant subject of their attention, were disposed to view hypochondria as but a form of insanity, regarded the pathology of the two as identical: but we shall merely observe that such identity is far from being established. We cannot perceive that M. Brachet, in his voluminous treatise, says anything about the morbid anatomy of hypochondriasis, probably for the satisfactory reason, that there was nothing precise or conclusive to be said.

*Treatment.* M. Brachet devotes a considerable portion of his work (from p. 460 to the end) to what he calls the "Therapeutical history," and the Therapeutics of hypochondria. The reader may anticipate that the pathology and morbid anatomy of the disease being so obscure and undefined, the treatment of it will be very vague and general and various: and a survey of the therapeutical methods suggested by the very numerous authors who have, during two or three centuries, written on the disease, will amply verify this anticipation. The details of treatment into which the author enters, whether by way of comment upon that of others, or as recommended by himself, are most minute and extensive; but we think that we are justified in making upon them the general remark, that they are equally applicable to almost any given disease whatever as to hypochondria. In fact, M. Brachet goes over nearly the whole field of hygiene and therapeutics in general, and brings it to bear on the disease: hence the reader, amid such vast details, loses sight of any *special* bearing which they have (if they have any such) on hypochondria. Both Georget and Falret's prospects of treatment are not very encouraging. "Hygienic measures," says the former, "furnish the principal and often the sole therapeutic resources." "I do not mean to say," observes the latter, "that we are to abstain from every species of medicine." "Place your whole reliance," he elsewhere remarks, "on the cerebral treatment, and on a wisely arranged regulation of the patient's life: recommend the use of simple aliments, soothing and easy of digestion. Proscribe aromatics." This is surely very *dilute* therapeutics, if we may be allowed to use the phrase. He afterwards gives his opinion of the expediency of bitters, chalybeates, tonics, purgatives, which we shall notice in due course.

In the department of the work now under notice, namely, the therapeutic, a considerable space is occupied in "enumeration and examination of the means advised against hypochondria." (p. 508 et seq.) Soothing treatment is that first noticed, and the mere names of the five classes into which Brachet divides it, will suggest to the reader the nature of it. Those are: 1st, temperant or diluent; 2d, mucilaginous or gummy; 3d, acidulous; 4th, analeptic, consisting of decoctions of veal, fowl, frogs, &c.; 5th, oleaginous. We shall not pause to make a single remark on this section (p. 514,) since there is nothing in it which calls for observation.

The expediency of milk as a diet in the disease is discussed in a separate section at p. 525. Hoffman, Cheyne, Tissot, Villermay, Dubois highly recommend it. The author himself, while remarking that it may be occasionly useful, and that its suitableness or unsuitableness can only be determined by careful and *prolonged* trials of the substance, in various forms and at different times of the day, makes the following very judicious practical observations in regard to this kind of diet, which we can fully confirm from practical observation. "In other circumstances, it (milk) weighs on the stomach, which has difficulty in passing it into the duodenum; it there acquires even acid and sharp qualities which cause pains and weight, flatus and nausea. In this case, milk is no longer soothing; but it is not irritating by its physical and intrinsic qualities; it only becomes so from its deficiency of stimulating power on the organ of digestion, which, instead of digesting, remains in a state of torpor." (p. 527.) In connexion with the same substance and subject, we quote the following remark from the succeeding page, on account of its general applicability to all cases in which the expediency of a milk diet may be a matter of consideration. "We agree with Sauvages in regarding milk as contra-indicated, in pituitous hypochondria, when the mucous membranes have become the seat of an abundant mucous secretion, conjoined to general debility; in all cases where there is idiopathic feebleness, independent of any organic lesion or of an ataxic nervous irritation of which it might be the consequence."

Antiphlogistic treatment is discussed at p. 542. Bloodletting, of course, occupies the first place, under this head. And on the principles and effects of bloodletting, the author's observations are exceedingly concise, just and perspicuous. He properly observes that in case of suppressed hemorrhoids, bloodletting by means of leeches is indicated: and that of course, any symptom leading to suspicion of cerebral congestion demands emission of blood either general or local. He also reasonably observes that "when an hypochondriac is young, of a good constitution, and of the sanguine temperament, when, above all, the malady is recent, bloodletting may be more useful and less hurtful." (p. 550.)

Soothing (calmante) treatment is next discussed. Under this head are comprised, hypnotics, anodynes, sedatives, narcotics. This species of treatment has, according to the author, three actions on the economy, a gastric, a nervine, and a cerebral. His remarks on this class of drugs are few and not very satisfactory. Opium, he believes, to be sometimes useful. For our own parts, the hypochondriac cases are very rare indeed, in which we should think of resorting to it. M. Brachet very properly remarks, after reminding us that hypochondriacs are "ordinarily tormented by an obstinate constipation" (p. 555,) "that opium, by diminishing the intestinal secretions and rendering sluggish their muscular contractions, augments instead of diminishing this constipation." And adds justly, that this circumstance should always be kept in view, in prescribing this substance.

In the remarks on antispasmodic treatment (p. 557,) and on the loadstone (p. 567) we find nothing to detain ourselves or the reader. At p. 569, we have some observations on animal magnetism and on somnambulism. Part of these we must be permitted to quote, were it for no other reason than for the author's amusing vivacity of expression.

"From the magnetism of Mesmer has gone forth animal magnetism. This second jugglery, twenty times prostrated by science, reason, and facts, lifts anew from time to time, its head, more amusing and more ridiculous than dangerous. We do not, however, deny the effects which the passes and other magnetic grimaces may produce on a person of a very nervous hysteric or hypochondriac constitution............We can conceive the possibility of the thing; cases of cure are cited. We do not, however, know any such which has been verified. Hence we do not regard this magnetism, even freed from all its parade of charlatanism, as a means on which we ought to reckon in hypochondria. We think, on the contrary, it is much more likely to be hurtful.

"If we observe some sort of reserve in regard to magnetism, we shall not do the like in regard to somnambulism, in connexion with which we have never seen any but rogues and dupes, and sometimes those burlesque imaginations, which long to fix the public attention, and to be the subject of its discourse at any price. Those distant journeys made without the person quitting his chair, those divinations, those transpositions of the senses, &c., are farces invented to amuse idlers and entrap fools. It may, however, chance that a weak and hypochondriac patient, strongly prepossessed in favour of this culpable jugglery, may derive from it some good effects; but then it is because he shall have been himself the dupe of his imagination and credulity, and not because he shall have been the subject of any real influence." (p. 570.)

Electricity is considered by M. Brachet as rather adapted to cases in which "the nerves are in a state of debility and atony near to paralysis, than when they are in a condition of too great erethism." (p. 573.)

We have several pages on the advantages to be derived from music in hypochondria. In selecting airs adapted for soothing the patient, we are directed (p. 576,) "to choose those which are most to his taste, which he loves most, and which, therefore, can best recall his imagination to its normal rhythm; for we must not think that gay airs are always those which will best succeed. The heavy and monotonous *Ranz des Vaches,* and the still more heavy *God save the King,* sound as agreeably to the ear of the Switzer or the Englishman, as to that of the Frenchman do "Vive Henri Quatre," or "Allons Enfants de la Patrie," &c.

*Tonic treatment.* Falret, we have already observed, was opposed to the use of chalybeates and bark in hypochondria. As Brachet justly remarks that debility is "rarely idiopathic or essential," and he judiciously cautions the practitioner not "to allow himself to be imposed upon by apparent debility; that he must not forget that nervous mobility and irritability are usually so great, as that he cannot be two distrustful of them," (i. e. tonics, more particularly chalybeates;) "that he must examine carefully the state of the circulation; an artery hard and tense, too much plasticity of the blood, contraindicate" tonics. (p. 583.) He directs particular attention to the state of the bowels, previous to the use of tonics; and most justly adds, in conclusion, "it must not be concealed, the cases in which tonics will do good are very rare." The cases in which chalybeates will be found useful, are those in which hypochondria occurs in "a constitution deteriorated, cacochymous, lymphatic, and with impoverished blood." M. Brachet, in such cases, advises their conjunction, with aromatics or bitters, such as canella, gentian, sarsaparilla, rhubarb, &c." (p. 586.)

M. Brachet's comments on "the treatment recommended against hypochondria," extend further through the following heads: evacuant, vomitive, purgative, sudorific, revulsive and derivative treatment; treatment by mineral waters, baths, injections, the trepan; moral and hygienic treatment; exercise and travel. Among all these, we would ourselves place most faith in a judicious system of mild purgative treatment, not too long continued, and of a course of mineral waters, with, it need not be added, due attention to exercise, diet, and moral recreation and regulation. In many cases of hypochondria, mild purgatives combined with tonics, are indicated. Indeed we have seldom seen a case in which they were not. Purgatives, as we have just observed, are neither to be given by themselves, nor are they to be so long continued as to debilitate: they must be combined with tonics. The report of the patient as to the regularity or condition of the alvine discharges must not be trusted to. The physician must satisfy himself on that head, and it is surprising how widely, on many occasions, his own observations will differ from those of the patient.

At page 646, M. Brachet lays down his own system of treatment, which he entitles "Methodical treatment," and which he thus divides: 1, Treatment of simple or slight hypochondria; 2, of chronic and constitutional hypochondria; 3, treatment of predominant symptoms; 4, treatment of the causes of hypochondria; 5, treatment of its complications; 6, treatment of convalescence and relapses; 7, prophylaxis.

From the comments which the author makes on the treatment of others, and which we have gone over at some length, a fair idea may be formed of M. Brachet's own plan. We shall not therefore travel through it. Under the head of "treatment of predominant symptoms" (p. 692, et seq.) he advises in gastralgia and gastrodynia, the hydrochlorate of morphia, combined with datura stramonium; he also names, as useful, belladonna, the cyanuret of potassium, aconite, &c. If these and antispasmodics fail, vesicatories, the actual cautery briefly used, the moxa and caustics may be applied to the epigastrium. If there is pain in the intestines, cassia, manna, tamarinds, oil of sweet almonds, injections and baths, will be useful. In cardialgia, sedatives are to be combined with antispasmodics; the cyanic preparations with assafœtida, musk, &c. In cerebral pain, opiates cautiously used, valerian alone or combined, hot foot-baths, asarum in powder as a sternutatory, are to be had recourse to. In palpitations, the cyanic preparations are alleged by the author to hold "the first place," (p. 699,) and in his hands, digitalis combined with the hydrochlorate of morphia, has produced the most unexpected results. "In *insomnia,* to which hypochondriac patients are very subject, opium given internally, is to be avoided as much as possible on account of its constipating effects; but from plasters and ointments of the drug, the author has obtained good results. He also advises that the different tinctures of opium should be applied, in friction, to the head. Against *flatus* of the stomach and bowels, one of the most characteristic inconveniences of hypochondria, Brachet recommends absorbents, &c., as magnesia, magistery of bismuth, crabs'-eyes, lime-water, soap, &c. Where the flatus depends on an *atonic* state of the stomach, M. Brachet recommends rhubarb, cascarilla, calumba, chamomile, cannella, anise, fennel, and even cinchona and chalybeates. In regard to constipation, an affection so frequent and troublesome with hypochondriasis, little is said.

The treatment of the *causes* of hypochondria, when these are appreciable, will, after what has been said, suggest itself to the reader. Into this point and the other particulars which we enumerated a few paragraphs back, the author enters, but in a very general and vague manner. Exercise, travel, recreation, occupation, and a certain degree of temporizing on the physician's part, are recommended. Attention to diet is of course strongly insisted on. In regard to prophylaxis, great care is suggested as necessary in regard to the habits, moral and physical, of youth, and exercise carried to *fatigue* is stated to be the physiological and safe way of forestalling, as it were, the operation of those passions, which at that early period often rage tempestuously, and the consequences of the unregulated operation of which are often experienced through the whole subsequent life.

We close this notice with the simple observation, that the work of M. Brachet must have obtained, in our opinion, the honour conferred on it by the French Royal Academy of Medicine, rather in consequence of the care and labour evinced in its production, than from any original merit to which it can lay claim. It must be allowed, however, to comprise a great amount of useful information on the subject it professes to treat.

II. Since the preceding pages were written, Dr. Michéa's 'Practical, Dogmatic, and Critical Treatise on Hypochondria' has been given to the public. This work, like the one we have just examined, was "crowned" by the French Royal Academy of Medicine. As the subject is the same, so the method and execution of Dr. Michéa's work are nearly identical with those of Brachet. Michéa's cases are, if we may so express ourselves, more picturesque; that is, many of them are extremely curious, both in a physical and in a metaphysical point of view. His "observations" (cases) amount to 81, and occupy fully one half of the volume. We shall give the "general reflections" (p. 262,) with which he sums up these cases.

"When one examines, not now in detail, but in a general view, the cases which we have given, we see that they include two groups of elements: symptoms which are constant and common" (to all cases,) "slight differences being excepted, and phenomena which are variable, existing in some cases and being absent in others. The symptoms constantly present are, the psychological disorder, the mental aberration; the varying characters are the functional alterations of the peripheric nervous system, and the lesions of tissue in the principal viscera." This distinction is important, for the order in which these two groups of symptoms appear, and their connexion can alone inform us whether hypochondria is an essential or a secondary affection, &c.

In Michéa's opinion, hypochondria is essentially *psychological* in its origin. We shall allow him to state the doctrine in his own words: (p. 369.)

"Now, in like manner, as religious monomaniacs which flow from the innate sentiment of deity (?) as eroto-mania which arises from the necessity of attachment proper to each sex; as the monomania of ambition, which is connected with the desire of elevation with which man is pursued, &c. &c.; so hypochondria connects itself very closely with a special faculty, with *the instinct of individual preservation*, the love of one's existence, the *biophilie* of Broussais ........The exaggeration of the faculty of which we speak, may be acquired or original."

And he thus comments on M. Brachet's view of the nature of the disease:

"M. Brachet, who prefers the opinion of Louyer Villermay to that of Willis, of Sauvages, of Darwin, of Cabanis; who seeks to refute the metaphysical assertion, which we regard as the principle, the germ of the malady in question; M. Brachet is extremely embarrassed, when he wishes to explain why pain does not always react on the encephalon, on the imagination, in such a manner as to produce hypochondria. For if the fear of suffering plays such an important part, as he thinks it does, it ought necessarily to follow, that the more an individual experiences a lively and protracted physical pain, the more he is apt to contract hypochondria. . . . . Also, not finding an explanation" [why such is not the case,] "neither in the diversity of the physical lesions peculiar to the nerves, nor in the peculiar character of the pain, M. Brachet is obliged to recognize a predisposition to hypochondria without specifying its nature; to see an enigma where none exists for him who knows how to examine things in a suitable manner, that is to say, to survey the question under all its aspects, under its moral as well as under its physical point of view. To recapitulate; the principle of hypochondriacal monomania, its virtual condition, the element without which all the properly named causes would be insufficient, and of non-effect, is a certain *exaggeration of the instinct of self-preservation*, in other words, of the *love of life*.

"The exaggeration of the faculty in question, may be *acquired* or *original*."

The treatment recommended by M. Michéa is so practically similar to that advised by M. Brachet, and by preceding authors, and presents so few novelties worthy of remark, that we shall not trespass on the reader's time, by any minute details of it. He divides the treatment into that of idiopathic and that of secondary hypochondria. In the former, he employs principally hygienic or physiological means, such as exercise, travel, intellectual occupation, and the *contra-excitation*, if we may use the word, of various salutary and innocent moral emotions and attractions. In the latter, he has recourse, in addition to physiological means, to anti-spasmodics, tonics, antiphlogistic, diaphoretic treatment. M. Michéa's work is well worthy of perusal by the English reader.

---

